# Icariin Activates Autophagy via Down-Regulation of the NF-κB Signaling-Mediated Apoptosis in Chondrocytes

**DOI:** 10.3389/fphar.2018.00605

**Published:** 2018-06-06

**Authors:** Bobin Mi, Junqing Wang, Yi Liu, Jing Liu, Liangcong Hu, Adriana C. Panayi, Guohui Liu, Wu Zhou

**Affiliations:** ^1^Department of Orthopaedics, Union Hospital, Tongji Medical College, Huazhong University of Science and Technology, Wuhan, China; ^2^State Key Laboratory of Molecular Vaccinology and Molecular Diagnostics & Center for Molecular Imaging and Translational Medicine, School of Public Health, Xiamen University, Xiamen, China; ^3^Addenbrooke’s Hospital, University of Cambridge School of Clinical Medicine, Cambridge, United Kingdom

**Keywords:** icariin, tissue engineering, autophagy, apoptosis, chondrocytes

## Abstract

Osteoarthritis (OA) is a common chronic and degenerative joint condition that is mainly characterized by cartilage degradation, osteophyte formation, and joint stiffness. The NF-κB signaling pathway in inflammation, autophagy, and apoptosis plays a prominent role in the progression of OA. Icariin, a prenylated flavonol glycoside extracted from Epimedium, have been proven to exert anti-osteoporotic and anti-inflammatory effects in OA. However, the action mechanisms of its effect on chondrocytes have yet to be elucidated. In the present study, we demonstrated that the *in vitro* therapeutic effects of icariin on rat chondrocytes in a dose-dependent manner. We found that TNF-α induced the production of IL-1, IL-6, IL-12, reactive oxygen species (ROS), nitric oxide (NO), Caspase-3, and Caspase-9 in chondrocytes. We also provided evidence that TNF-α inhibited autophagy markers (Atg 5, Atg 7) and prevented LC3 I translate to LC3 II. Furthermore, TNF-α induced matrix metalloproteinase (MMP)3 and MMP9 expression. The negative effects of TNF-α on chondrocytes can be partially blocked by treating with icariin or ammonium pyrrolidinedithiocarbamate (PDTC, an NF-κB inhibitor). The present study data also suggested that icariin suppressed both TNF-α-stimulated p65 nuclear translocation and IκBα protein degradation. These results indicated that icariin protected against OA by suppressing inflammatory cytokines and apoptosis, through activation of autophagy via NF-κB inhibition. In conclusion, icariin appears to favorably modulate autophagy and apoptosis in chondrocytes making it a promising compound for cartilage tissue engineering in the treatment of OA.

## Introduction

Osteoarthritis (OA) is a chronic degenerative joint disease that occurs mostly in the elderly (Glyn-Jones et al., 2015). As the population ages, the occurrence of OA increases and, consequently, finding an effective treatment is imperative. The capacity of chondrocytes to regenerate normal cartilage matrix architecture declines with aging, resulting in cartilage degradation and erosion (Blanco et al., 2011). In the cases of OA, various changes occur in the cartilage including inflammation, oxidative stress, loss of cartilage matrix, autophagy, and apoptosis.

During the progression of OA, the increased expression of inflammatory cytokines (such as TNF-α and IL-1β) in articular cartilage and synovium contribute to the degradation and erosion of cartilage (Raman et al., 2018). At the same time, autophagy and apoptosis occur in the progression of OA. Autophagy is necessary for maintaining the cell’s metabolism and homeostasis, and for cellular quality control by clearing waste or damaged proteins and organelles (Galluzzi et al., 2014). The dysregulation of autophagy that happens with OA contributes to the degeneration of the articular cartilage (Vasheghani et al., 2015). The importance of autophagy in preventing age-related OA has been demonstrated by the increasing number of studies on the topic (Duarte, 2015; Loeser et al., 2016). In addition, more and more evidences suggest that increased chondrocyte apoptosis induces cartilage degeneration in OA (Kobayashi et al., 2016). Thus, activation of autophagy and inhibition of apoptosis in chondrocytes may limit OA progress.

Recently, various biomaterials, such as hydrogels, have been used as drug delivery systems to regulate chondrocyte autophagy (Chen et al., 2016). Driven by the rapid progression of nanomedicine and nanotechnology (Tao et al., 2015, 2017a; Ding et al., 2017; Rosenblum et al., 2018), compounds have been increasingly studied in the context of regeneration medicine and tissue engineering. However, few compounds are reported to be useful for tissue engineering in cartilage repair. Icariin is a well-known compound extracted from Herba Epimedil, with a wide range of pharmacological effects, including anti-inflammatory, anti-atherosclerotic, and anti-oxidative properties (Fang and Zhang, 2017; Wang G.Q. et al., 2017; Xiong et al., 2017). Recently, icariin-mediated chondroprotective effects have attracted growing attention. Icariin protects against OA by inhibiting overexpression of metalloproteinase 13 (MMP-13) and proinflammatory cytokines in chondrocytes (Zeng et al., 2014). In addition to inhibiting H_2_O_2_-induced human umbilical vein endothelial cell apoptosis, icariin suppresses NF-κB signaling in macrophages (Wang and Huang, 2005; Chen et al., 2010). The effect of icariin on chondrocyte autophagy and apoptosis, however, remains unclear.

In this study, we investigate whether icariin has chondroprotective effects against TNF-α-induced cell death. These effects might be closely related with autophagy activation and apoptosis inhibition. In addition, we explore the underlying mechanisms of icariin-mediated cell autophagy and apoptosis.

## Materials and Methods

### Reagent, Antibodies, and Ethics Statement

Icariin was purchased from Sigma-Aldrich (MO, United States). Cell Counting Kit-8 (CCK8) was purchased from MedChemExpress (NJ, United States). Antibodies against ATG5, ATG7, LC3, p65, phosphorylated p65 (p-p65), IκBα, MMP3, MMP9 were purchased from Abcam (Cambridge, United Kingdom). Antibody against GAPDH was purchased from Cell Signaling Technology, Inc. (MA, United States). The NF-κB inhibitor pyrrolidinedithiocarbamate (PDTC) was purchased from Abcam (Cambridge, United Kingdom). Enzyme-linked immunosorbent assay (ELISA) kits were purchased from Bio-Swamp Life Science (Shanghai, China). The Sprague-Dawley (SD) rat was purchased from The Center of Experimental Animal, Tongji Medical College, Huazhong University of Science and Technology. All experimental procedures were approved by the Institutional Animal Care and Use Committee at Tongji Medical College, Huazhong University of Science and Technology.

### Cell Isolation and Culture

Chondrocytes were isolated from the articular cartilage of 7–10-day-old male SD rats. Briefly, pieces of articular cartilage were digested with 0.25 mg/mL trypsin for 30 min and 2 mg/mL collagenase type II for 8 h at 37°C. After digestion, isolated chondrocytes were passed through a 180-μm filter and the cells were centrifuged and washed with PBS several times. The cells were then isolated and stained with trypan blue to evaluate cell viability. Chondrocytes with viability greater than 85% were cultured in Dulbecco’s modified Eagle’s medium (DMEM) containing 10% fetal bovine serum (FBS) and 100 IU/ml penicillin, 100 μg/ml streptomycin at 37°C, 5% CO_2_. When the cells were treated with 50 ng/ml TNF-α, 10 μM icariin, 10 μM PDTC, icariin or PDTC were added to the medium 2 h prior to TNF-α addition. In the autophagic flux assay, 0.1 μm bafilomycin A1 was added to the medium 1 h prior to icariin or PDTC addition. In the experiments, chondrocytes were treated with PBS (control), TNF-α, icariin with TNF-α, or PDTC with TNF-α for 24 h. All experiments were conducted in triplicate.

### Cytotoxicity Assay

Rat chondrocytes were seeded in 96-well plates (5 × 10^3^ cells/well) overnight, followed by treatment with various concentrations of icariin for 24 h, 48 h, and 72 h at 37°C in an atmosphere containing 5% CO_2_. Following this, 10 μl CCK8 solution was added to each well and the cells were cultured at 37°C for 2 h. The OD value was then measured with a microplate reader (Thermo Fisher Scientific, United States) at 450 nm.

### ELISA

Chondrocytes were seeded onto a 24-well plate at 2 × 10^4^ cells per well. Following 24 h incubation, cells were treated with PBS, TNF-α, icariin with TNF-α, or PDTC with TNF-α. The supernatants were collected after 24 h incubation and the levels of IL-1, IL-6, and IL-12 were quantified using the ELISA kits.

### Western Blot

Total proteins were extracted using cold RIPA buffer containing protease inhibitor (Boster Biological Technology, Ltd., Wuhan, China). Proteins were quantified using the bicinchoninic acid protein assay kit (BCA kit) according to the manufacturer’s instructions. Equal amount of proteins (10 μg) were separated by 10% SDS-PAGE and transferred to polyvinylidene fluoride (PVDF) membranes (Millipore, United States). Membranes were blocked in 5% bull serum albumin (BSA, Sigma, United States) in TBST for 2 h, then incubated with primary antibody at 4°C overnight. The primary antibodies were as follows: p65 (1:2000), p-p65 (1:2000), IκBα (1:5000), ATG5 (1:5000), ATG7 (1:10000), LC3 (1:3000), MMP3 (1:10000), MMP9 (1:10000), and GAPDH (1:1000). After washing with TBST (50 mM Tris PH 8.0, 150 mM NaCl, 0.01% Tween-20) three times, the membrane was incubated with HRP-conjugated corresponding secondary antibodies (Goat Anti-Rabbit IgG, 1:10000) for 1 h at room temperature. Following washing, the immunoreactive proteins were visualized with the ECL western detection kit (Thermo Fisher Scientific, United States). The band density was quantified using TANON GIS software (Tanon, Shanghai, China).

### Reverse Transcription-Quantitative (RT-q) PCR

Total RNA from chondrocytes was extracted using Trizol reagent (Thermo Fisher Scientific, United States). Total RNA was reverse-transcribed to cDNA with RT Master Mix (Takara Japan). The RT-PCR was performed with SYBR Master Mix using StepOne-Plus system (ABI, United States) under the following conditions: denature at 95°C for 30 s, anneal at 60°C for 1 min and extend at 95°C for 5 s. The gene expression was analyzed by 2^-ΔΔCt^ method using GAPDH as the internal control. The primer sequences were as follows: ATG5, forward (5′-AA CGAGAAGCAGAGCCA-3′) and reverse (5′-ATGCCAT TTCAGGGGTG-3′); ATG7, forward (5′-GAAGAACCAGAAA GGAGG-3′) and reverse (5′-CAGGCACTTGACAGACAC-3′); Bax, forward (5′-TGGTTGCCCTCTTCTA-3′) and reverse (5′CACCCTGGTCTTGGAT-3′); Bcl-2 forward (5′-CACAG AGGGGCTACGAGT-3′) and reverse (5′-CAGGCTGGAAGG AGAAGA-3′); GAPDH, forward (5′-CAAGTTCAACGGCA CAG-3′) and reverse (5′-CCAGTAGACTC CACGAC AT-3′).

### Transmission Electron Microscopy (TEM)

Collected cells were fixed with 2.5% glutaraldehyde for 24 h, which followed by fixed with 1% osmium tetroxide for 1 h at 4°C. After dehydrated with a series of ethanol concentrations (50, 70, 80, 90, and 100%) for 10-min intervals, the samples were incubated in a mixture of acetone and epoxy resin (v:v = 1:1) for 6 h, followed by incubation with pure epoxy resin for 4 h. After semi-thin sectioning, cells were stained with 0.5% toluidine blue and observed under the microscope. Finally, the ultra-section sections were observed using a TEM (Hitachi, Japan).

### Cell Cycle Assay

Collected cells were washed and suspended in 0.3 mL PBS containing 10% FBS and 0.7 mL ethyl alcohol for 24 h at -20°C. The cells were then suspended with 0.1 mL RNase A (1 mg/mL) and 0.4 mL propidium iodide (PI) (50 μg/mL) for 10 min. The percentage of cells in the different stages was measured using Flow Cytometry.

### Cell Apoptosis Assay

Collected cells were stained with a mixture of calcein-AM and PI solution for 20 min. Fluorescence images of cells were then recorded using an inverted fluorescent microscope. The percentages of cell death were evaluated by calculating the number of PI-stained (dead, red) and calcein-AM-stained (live, green) cells. The number of cells were counted in five random fields by three independent authors. The mean value of each measurement was used for analysis.

### Measurement of ROS Production

The ROS level was measured using Reactive oxygen species assay kit (Nanjing Jiancheng Bioengineering Institute, Nanjing, China) according to the manufactures’ instructions. Briefly, treated cells were incubated with 10 μM dichlorodihydrofluorescein diacetate (DCFH-DA) in the dark for 30 min. Then, the cells were rinsed with DMEM and analyzed on a flow cytometer with excitation wavelength of 500 nm and emission wavelength of 525 nm. ROS level in the experimental group was normalized to the control group.

### Measurement of NO Production

NO production was detected with nitrate/nitrite colorimetric assay kit of Griess reaction (Nanjing Jiancheng Bioengineering Institute, Nanjing, China) according to the manufacturer’s protocol. In brief, cultured medium was collected from treated cells and mixed with equal volume of Griess reagent. Following a 10-min incubation, absorbance was measured at 550 nm on a microplate reader (Thermo Fisher Scientific, United States).

### Caspase-3 and Caspase-9 Activity Assay

Caspase-3 and caspase-9 was detected with Caspase colorimetric assay kit (Nanjing Jiancheng Bioengineering Institute, Nanjing, China). Briefly, collected cells were lysed in the provided lysis buffer. The absorbance was measured at 405 nm on a microplate reader (Thermo Fisher Scientific, United States).

### Immunocytochemical Staining of p65

Cells were collected and washed with PBS, and then fixed with 4% paraformaldehyde for 15 min. After washing with PBS, the cells were blocked with 5% BSA for 2 h at 37°C. Then, p-p65 antibody (1:150) was added and incubated overnight at 4°C, followed by adding Max Vision TMHRP-polymer for 1 h, and followed by incubation with DAB for 5 min. The number of p65 in the nucleus was counted in five random fields by three independent authors. The mean value of each measurement was taken for analysis.

### Statistical Analysis

Data were presented as means ± standard deviation (*SD*). Statistical analysis was performed in GraphPad Prism 6.0 (GraphPad Software, United States) using Student’s *t*-test and one-way analysis variance (ANOVA). Statistical significance was considered when *P* < 0.05.

## Results

### Cytotoxicity of Icariin on Chondrocytes

**FIGURE 1:**
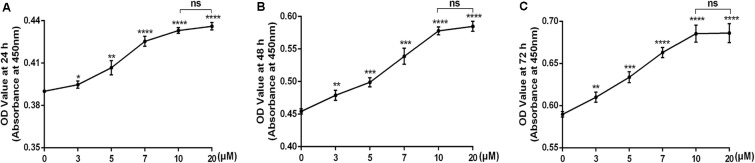
**(A–C)** The cytotoxicity of icariin on chondrocytes was examined using the concentration of 0, 3, 5, 7, 10, and 20 μM after 24, 48, and 72 h of culture. Values were expressed as means ± SD. ^∗^*P* < 0.05, ^∗∗^*p* < 0.01, ^∗∗∗^*p* < 0.001, ^∗∗∗∗^*p* < 0.0001.

To determine whether icariin was toxic to chondrocytes, CCK8 assay was used after the cells had been treated with increasing concentration of icariin (0, 3, 5, 7, 10, 20 μM) for 24, 48, and 72 h. As shown in **Figure 1**, icariin promoted cell viability in a dose-dependent manner. The dose of 10 μM and 20 μM icariin had a similar beneficial effect on chondrocytes. (**Figure 1**) The dose of 10 μM was selected for the subsequent experiments.

### Effects of Icariin on TNF-α-Induced Inflammatory Cytokines

**FIGURE 2:**
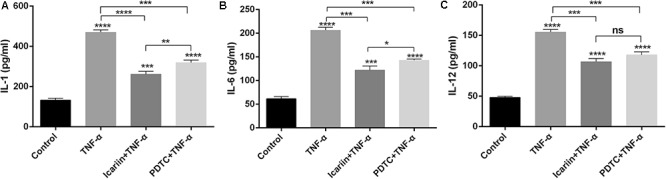
Effect of PBS, TNF-α, icariin with TNF-α, and PDTC with TNF-α on the production of inflammatory cytokines. **(A–C)** TNF-α induced the production of IL-1, IL-6, and IL-12 in chondrocytes. Icariin or PDTC partially attenuated the TNF-α-induced inflammatory cytokines production. Values were expressed as means ± SD. ^∗^*P* < 0.05, ^∗∗^*p* < 0.01, ^∗∗∗^*p* < 0.001, ^∗∗∗∗^*p* < 0.0001.

There are various inflammatory cytokines involved in the pathologic process of OA. These cytokines, such as IL-1β, induce the production of the other inflammatory cytokines (Hong et al., 2014), leading to inflammatory milieu in chondrocytes. In the present study, we found that TNF-α induced the production of IL-1, IL-6, and IL-12 in chondrocytes. Such effects were partially blocked with the addition of icariin to TNF-α-treated cells (**Figure 2**), suggesting that icariin had an anti-inflammatory effect on chondrocytes.

### Icariin Activates Autophagy in TNF-α-Treated Chondrocytes

**FIGURE 3:**
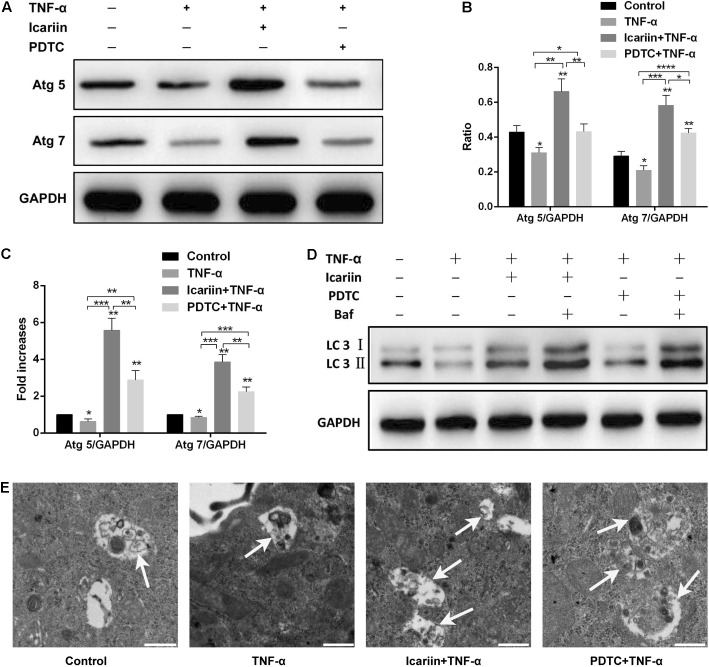
Effect of PBS, TNF-α, icariin with TNF-α, and PDTC with TNF-α on the autophagy of chondrocytes. **(A–D)** Western blot and PCR analysis indicated that icariin and PDTC could partially reverse the TNF-α-induced inhibition of autophagy. Bafilomycin A1 significantly increased the LC3- II level in icariin-TNF-α-treated cells and in PDTC-TNF-α-treated cells. **(E)** Transmission electron microscopy from four groups. Autophagosomes in images are marked with black arrows. White arrow represents autophagosome in the cytoplasm. Scale bar = 500 nm. Values are expressed as means ± SD. ^∗^*P* < 0.05, ^∗∗^*p* < 0.01, ^∗∗∗^*p* < 0.001, ^∗∗∗∗^*p* < 0.0001. Baf: Bafilomycin A1.

To investigate the effect of icariin on the autophagy of chondrocytes, the autophagy markers Atg 5 and Atg 7 were detected. The western blot and PCR results showed that autophagy markers were significantly reduced in TNF-α-treated cells when compared with those cells co-treated with icariin and TNF-α, indicating that icariin protected against TNF-α-induced inhibition of autophagy (**Figures 3A–C**). In addition to Atg 5 and Atg 7, western blotting result of LC3 (a classic marker of autophagy) was also used to establish the effect of icariin on autophagy. Icariin significantly enhanced LC3-II level when adding to the TNF-α-treated cells (**Figure 3D**). During the late phase of autophagy, the fusion of lysosomes with autophagosomes may lead to a lower accumulation of LC3-II in the cytoplasm. To demonstrate this effect, bafilomycin A1 was added 24 h prior to cells co-treated with TNF-α and icariin to inhibit the fusion of lysosomes with autophagosomes. The level of LC3-II increased when bafilomycin A1 was added to the TNF-α-icariin-treated cells (**Figure 3D**), suggesting that autophagy may fail to detect when autophagosomes fusion with lysosomes. Furthermore, TEM images illustrated that the number of autophagosomes in cells co-treated with icariin and TNF-α was higher than that cells treated solely with TNF-α (**Figure 3E**). These results indicated that icariin activated autophagy in TNF-α-treated chondrocytes.

### Effects of Icariin on TNF-α-Induced Proliferation Inhibition and Apoptosis Activation

**FIGURE 4:**
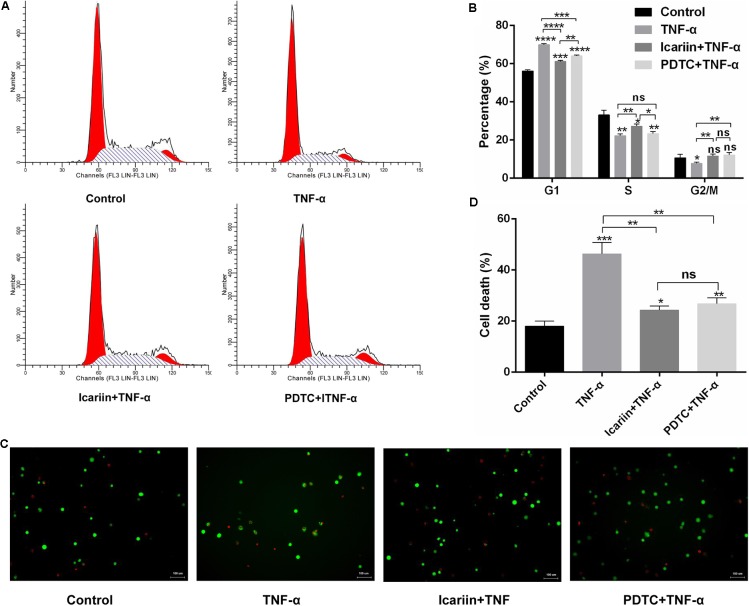
Effect of PBS, TNF-α, icariin with TNF-α, and PDTC with TNF-α on cell cycle progression and apoptosis of chondrocytes. **(A)** The distribution of cell cycles after 24 h treatment. **(B)** TNF-α induced cell cycle arrest at the G1 phase. Icariin or PDTC induced TNF-α-treated cell cycle arrest at the S-G2/M phase. **(C)** Calcein-AM/PI double-staining of apoptotic cell death. **(D)** Analysis of apoptosis revealed that TNF-α induced chondrocyte apoptosis. Icariin or PDTC partially blocked the TNF-α-induced chondrocyte apoptosis. Magnification: 200×. Values are expressed as means ± *SD*. ^∗^*P* < 0.05, ^∗∗^*p* < 0.01, ^∗∗∗^*p* < 0.001, ^∗∗∗∗^*p* < 0.0001.

It is well known that inhibition of the proliferation rate correlates with decreased cell cycle progression in chondrocytes. We analyzed the cell cycle division of chondrocytes treated with PBS, TNF-α and icariin with TNF-α. According to the PI staining of flow cytometry analysis, TNF-α arrested chondrocytes in the G1 phase. However, the percentage of the cell population in the S phase increased from 22.27% to 26.11% when addition of icariin to the TNF-α-treated chondrocytes (**Figures 4A,B**). These results suggested that cell cycle progression might be delayed by TNF-α, a delay that is partially reversed by icariin treatment.

**FIGURE 5:**
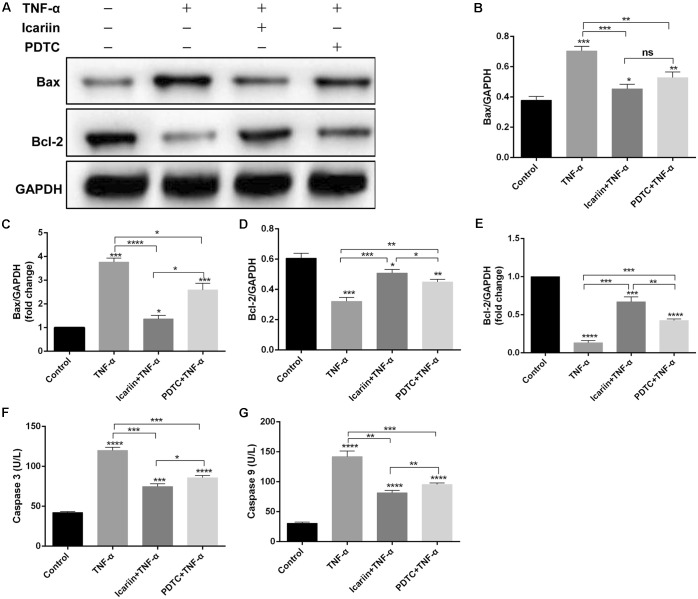
Effect of PBS, TNF-α, icariin with TNF-α and PDTC with TNF-α on the apoptosis of chondrocytes. **(A–E)** Western blot and PCR analysis showed that the TNF-α group had a higher level of pro-apoptotic protein Bax and a lower level of anti-apoptotic protein Bcl-2 compared with the other three groups. **(F,G)** ELISA results showed that the level of Caspase-3/9 was higher in the TNF-α group than the other three groups. Values were expressed as means ± *SD*. ^∗^*P* < 0.05, ^∗∗^*p* < 0.01, ^∗∗∗^*p* < 0.001, ^∗∗∗∗^*p* < 0.0001.

We also analyzed the effect of TNF-α on chondrocyte apoptosis by calcein-AM/PI double-staining and found that TNF-α significantly induced chondrocyte apoptosis. However, when icariin was added to TNF-α-treated cells, the percentage of cell death decreased from 46.33% to 24.33% (**Figures 4C,D**), suggesting that icariin protected against TNF-α-induced cell apoptosis. To further determine whether icariin affects the mitochondrial apoptosis pathway of chondrocytes, the anti-apoptotic protein Bcl-2 level and the pro-apoptotic Bax level were detected using western blot and PCR. TNF-α significantly inhibited Bcl-2 expression but induced Bax expression. These effects were partially blocked by icariin treatment (**Figures 5A–E**). Furthermore, the effect of that TNF-α-induced Caspase-3/9 activation in chondrocytes was blocked by addition of icariin. (**Figures 5F,G**).

### Effect of Icariin on TNF-α-Induced NO and ROS Production and Catabolism in Chondrocytes

**FIGURE 6:**
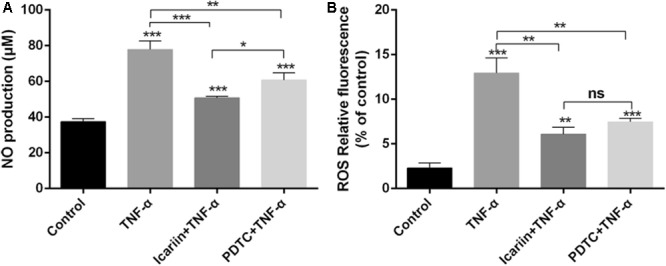
Effect of PBS, TNF-α, icariin with TNF-α, and PDTC with TNF-α on the production levels of NO and ROS. **(A,B)** TNF-α induced the production of NO, ROS, an effect which could be reversed by adding icariin or PDTC treatment. Values were expressed as means ± SD. ^∗^*P* < 0.05, ^∗∗^*p* < 0.01, ^∗∗∗^*p* < 0.001.

The level of NO and ROS is known to increase when chondrocytes are exposed to inflammatory cytokines. In the present study, we found that TNF-α significantly induced NO and ROS production, an effect which could be blocked by addition of icariin (**Figure 6**).

**FIGURE 7:**
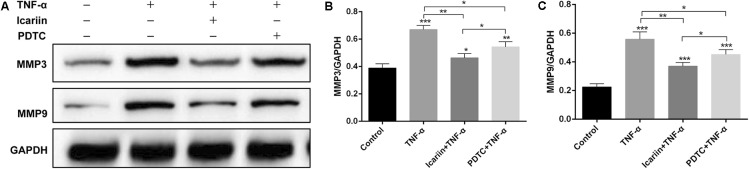
Effect of PBS, TNF-α, icariin with TNF-α and PDTC with TNF-α on the catabolism of chondrocytes. **(A–C)** Western blot analysis showed that the levels of MMP3 and MMP9 were higher in the TNF-α group than the other three groups. Values were expressed as means ± SD. ^∗^*P* < 0.05, ^∗∗^*p* < 0.01, ^∗∗∗^*p* < 0.001.

Disruption of homeostasis in cartilage metabolism impairs the extracellular matrix and chondrocytes, resulting in cartilage degradation. The excessive matrix catabolism was usually caused by excessive mechanical joint loading or inflammatory cytokines, which was reflected by the increased levels of matrix-degrading enzymes matrix metalloproteinase (MMP) family. In the present study, TNF-α induced the production of MMP 3 and MMP 9, an effect which could be blocked by addition of icariin (**Figure 7**). These results indicated that icariin inhibited NO and ROS production and reconstituted homeostasis of metabolism in chondrocytes.

### Icariin Inhibits NF-κB Pathway Activated by TNF-α in Chondrocytes

**FIGURE 8:**
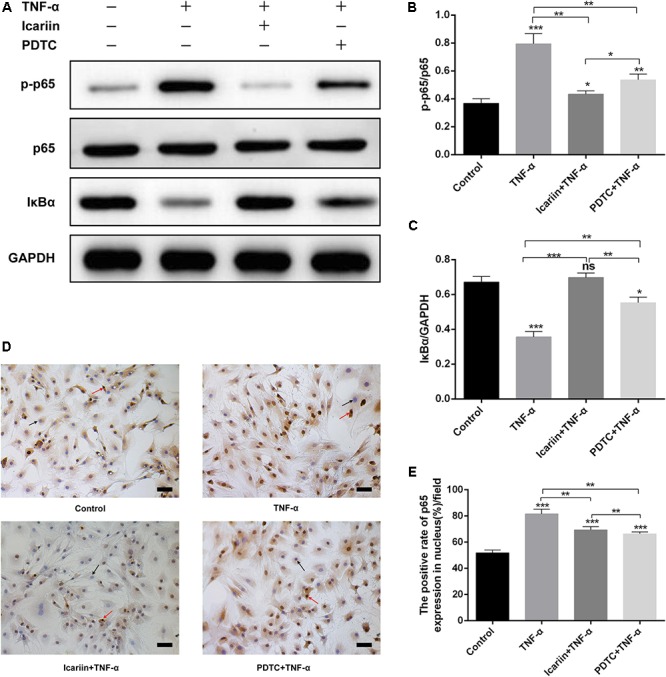
Effect of PBS, TNF-α, icariin with TNF-α and PDTC with TNF-α on the NF-κB signaling pathway. **(A–C)** TNF-α significantly increased the level of *p*-p65 and decreased the level of IκBα, an effect which could be partially reversed by adding icariin or PDTC treatment. **(D,E)** More p65 translocated to the nucleus in TNF-α-stimulated cells, a process which was blocked with the addition of icariin or PDTC treatment. The black arrow indicated nucleus and red arrow indicated p65 translocated to the nucleus. Values were expressed as means ± SD. ^∗^*P* < 0.05, ^∗∗^*p* < 0.01, ^∗∗∗^*p* < 0.001. Magnification: 200×. Scale Bar = 50 μm.

In the present study, western blot results showed that TNF-α significantly increased the level of p-p65 and decreased the level of IκBα in chondrocytes when compared with the control group, suggesting that TNF-α stimulation of the NF-κB pathway (**Figures 8A,B**). This finding also proven by the result of immunocytochemical staining that the number of p65 translocate to nucleus in TNF-α-treated cells is higher than the control group (**Figures 8C,D**). In contrast, icariin caused the TNF-α-induced p-p65 increase and IκBα degradation reversal. Furthermore, the number of p65 translated to the nucleus also decreased by addition of icariin to TNF-α-treated cells. (**Figure 8**).

### Involvement of NF-κB Pathway in the Autophagy Activation and Apoptosis Inhibition Induced by DHA

The NF-κB pathway plays a crucial role in various pathogenesis process of OA. To investigate whether this pathway was involved in the TNF-α-induced negative effect on chondrocytes, we used an NF-κB inhibitor, PDTC. In the present study, we found that PDTC partially blocked the TNF-α-induced inflammatory cytokines production, proliferation and autophagy inhibition, apoptosis activation, and hypercatabolism. (**Figures 2**–**8**).

## Discussion

Inflammatory cytokines including TNF-α and IL-6 contribute to cartilage catabolism and degeneration in OA (Lai et al., 2014; Nasi et al., 2016). Excessive inflammatory cytokines inhibit autophagy activation, further increasing production of ROS and leading to cell death (Liu-Bryan and Terkeltaub, 2015). In the current study, we demonstrated that TNF-α suppressed autophagy in chondrocytes, which was reflected by the lower level of autophagy marker. Previous studies found that TNF-α inhibited the expression of Atg 5 and suppressed the conversion of LC3 I to II (Jiang et al., 2016). With Atg 5 knockdown, mice were more likely to develop OA with aging (Bouderlique et al., 2016), suggesting that activation of autophagy has a beneficial effect in preventing OA. When inflammatory cytokines release, the level of ROS and NO production increases, ultimately leading to chondrocyte apoptosis (Akuri et al., 2017). The present study results consistent with previous studies which showed that TNF-α induced chondrocyte apoptosis via upregulated expression level of ROS and NO. In addition, previous studies, as well as the current study, have demonstrated that TNF-α induced the pro-apoptotic protein Bax production and reduced the anti-apoptotic protein Bcl-2 production, leading to hypercatabolism in chondrocytes (Ye et al., 2015). It should be noted that there are other proinflammatory cytokines contribute to the autophagy suppression and apoptosis induction in OA (Goldring and Otero, 2011). These cytokines may result in a synergistic effect in inhibiting autophagy and activating apoptosis during the pathological process of OA. Unfortunately, the present study reported that TNF-α further induced the production of other inflammatory cytokines. Therefore, it is necessary to regulate the inflammatory processes of OA. Indeed, treatments of OA with non-steroidal anti-inflammatory drugs that inhibit the release of proinflammatory cytokines is common practice (Urech et al., 2010).

The cytotoxic effects of icariin on other cell types have been investigated (Zhang et al., 2015). In the present study, icariin exhibited a positive effect on cell viability, in a dose-dependent manner. Both doses of 10 μM and 20 μM icariin showed similar effect on cell viability. Previous studies reported that icariin alleviated the inflammatory response in most cell types (Pan et al., 2017; Sun et al., 2018). In the present study, we demonstrated that icariin attenuated the production of inflammatory cytokines in chondrocytes. As overproduction of inflammatory cytokines inhibit autophagy activation (Qi et al., 2014), it is logical to suppose that icariin reverses the inflammatory cytokines-induced autophagy inhibition. Some studies reported that icariin plays a beneficial role on cell survival by inhibiting autophagy (Tang et al., 2015; Li et al., 2017). In the present study, however, we found that icariin blocked the TNF-α-induced autophagy inhibition in chondrocytes. This result may be attributed to its anti-inflammatory effect on chondrocytes. When autophagy activation, it could further suppress inflammatory response in chondrocytes (Ansari et al., 2017). Given that icariin and autophagy have both been shown to attenuate inflammatory response, this may act as a positive feedback loop to suppress TNF-α-induced inflammation. Along with the production of inflammatory cytokines was suppressed by icariin, so was apoptosis. In addition, the decreased level of NO and ROS induced by icariin also contribute to the inhibition of apoptosis and catabolism (Shen et al., 2015; Qiao et al., 2018). The apoptosis results were consistent with previous studies that support that icariin had protective effect against apoptosis (Deng et al., 2017). Consequently, icariin plays a dual role of autophagy activation and apoptosis inhibition in chondrocytes.

Previous studies reported that activation of the NF-κB signaling pathway induced pro-inflammatory cytokines release (Dubey et al., 2018). Exposure of chondrocytes to a variety of inflammatory cytokines lead to the degradation of IκB, allowing p65 translocate to the nucleus (Guo et al., 2015). The present study showed that TNF-α induced NF-κB pathway activation. Icariin is known to inhibit NF-κB signaling, leading to anti-inflammatory effect (Hua et al., 2018). Thus, the TNF-α-induced NF-κB activation was reversed with addition of icariin, suggesting that icariin had a negative effect on NF-κB activation. NF-κB plays a key role in both chondrocyte autophagy and apoptosis. When the NF-κB signaling pathway was activated by inflammatory cytokines, autophagy inhibition along with apoptosis activation appear, which play a synergetic effect on accelerating chondrocyte death (Jiang et al., 2016; Zhang et al., 2016). The results that autophagy markers significantly increased and apoptotic markers significantly decreased when addition of icariin or PDTC to the TNF-α-treated chondrocytes, suggested that icariin protected against OA by inhibiting the TNF-α-induced NF-κB signaling pathway activation. Interestingly, we noticed that icariin treatment had a better anti-inflammatory effect than PDTC treatment on TNF-α-treated cells in terms of IL-1 and IL-6. In addition, it also had a better reversal effect than PDTC, i.e., on the negative effect of TNF-α-induced autophagy inhibition and apoptosis activation. Previous studies reported that in addition to the NF-κB signaling pathway, other signaling pathways such as the MAPK/JNK and ERK pathways are also involved in the pathogenesis process of OA (Wang et al., 2018). In the future experiments, we would like to verify whether icariin protects against OA via these or other signaling pathways.

Tissue engineering is an evolving interdisciplinary field integrating medicine, material science, biochemistry, and biomedical engineering, which centered on development of biological alternates to restore and/or to improve tissue and organ function (Tao et al., 2013; Tao et al., 2016; Zhu et al., 2018). Along with the development of biomaterial, the drug delivery system has attracted more attention in the treatment of cancer, diabetes and OA (Tao et al., 2017b; Wang J. et al., 2017; Zhao et al., 2017; Maudens et al., 2018). Jiang et al. (2018) developed a poly (lactic-co-glycolic acid)-based nanoscale drug delivery system for the treatment of OA. Li et al. (2012) reported that icariin up-regulated the expressions of aggrecan, sox9, and collagen I of chondrocytes, features which make it a potential promoting compound for cartilage tissue engineering. Previous studies found that targeted microspheres loaded with icariin could exert colon-protective effects through reduction of the inflammatory response (Wang et al., 2016). Pan et al. (2016) also reported that icariin loaded biphasic-induced magnetic CS/nHA/MNP microcapsules is a useful drug delivery system for bone repair. Considering the various beneficial effects of icariin, it could be considered an excellent compound to be used in drug-delivery system. In the future studies, we plan to look for a good biocompatibility drug delivery system which is suitable to load icariin for the treatment of OA.

In conclusion, we demonstrated that icariin had no cytotoxic effects on chondrocytes up to the dose of 20 μM. TNF-α induced inhibition of autophagy and activation of apoptosis, and increased inflammatory cytokines, NO and ROS, as well as stimulating catabolism. These negative effects could be partially reversed by adding icariin to TNF-α-treated chondrocytes via inhibition of NF-κB signaling. Thus, the present study highlights that icariin induces autophagy activation and apoptosis inhibition of chondrocytes via suppression of the NF-κB signaling pathway. Future *in vivo* evaluation using macro-hydrogels to explore icariin’s therapeutic potential in OA treatment is currently under way in our laboratory.

## Author Contributions

BM, WZ, and GL conceived and designed the experiments. JL, YL, and LH performed the experiments. BM and JW wrote the manuscript and made the same contribution to the manuscript. AP revised the language of the manuscript.

## Conflict of Interest Statement

The authors declare that the research was conducted in the absence of any commercial or financial relationships that could be construed as a potential conflict of interest. The reviewers and handling Editor declared their shared affiliation.
